# Evaluation of *‘Shisha No Thanks’* – a co-design social marketing campaign on the harms of waterpipe smoking

**DOI:** 10.1186/s12889-022-12792-y

**Published:** 2022-02-24

**Authors:** Lilian Chan, Nouhad El-Haddad, Becky Freeman, Ross MacKenzie, Lisa Woodland, Blythe J. O’Hara, Ben F. Harris-Roxas

**Affiliations:** 1 Prevention Research Collaboration, Sydney School of Public Health and Charles Perkins Centre, The University of Sydney, 2006 Camperdown, NSW Australia; 2 Centre for Primary Health Care and Equity, Faculty of Medicine, University of New South Wales, Sydney, Australia; 3 Population and Community Health, South Eastern Sydney Local Health District, Darlinghurst, Australia; 4 School of Population Health, Faculty of Medicine, University of New South Wales, Sydney, Australia

**Keywords:** Waterpipe, Smoking, Tobacco control, Campaign, Social marketing

## Abstract

**Background:**

Waterpipe (shisha) is becoming increasingly popular worldwide, particularly among young people; and in some countries, it is one of the few forms of tobacco use that is increasing. While there is a growing body of evidence of the harms of waterpipe smoke, there is a scarcity of research of interventions to address this form of tobacco consumption.

**Methods:**

The *Shisha No Thanks* project was a co-design social marketing campaign that aimed to raise awareness of the harms of waterpipe smoking among young people from an Arabic speaking background in Sydney, Australia. The campaign distributed material through social media and community events. We evaluated the project through an SMS community panel using a longitudinal study design. The cohort were sent questions before and after the project asking about their awareness of messages of harms, attitudes, intention to reduce waterpipe smoking, and awareness of support services. Data was analysed as matched pre- post- data.

**Results:**

The evaluation recruited 133 people to the panel. There was a significantly greater proportion of people who reported seeing, hearing or reading something about the harms of waterpipe smoking after the campaign (67.5%) compared with before (45.0%) (*p*=0.003). Post-campaign, there were higher proportions of people who strongly agreed that waterpipe smoking causes damage, and that it contains cancer-causing substances, but these increases were not statistically significant. There was low awareness of waterpipe cessation services at baseline and post campaign (22.5%).

**Conclusions:**

The *Shisha No Thanks* project increased awareness of messages about the harms of waterpipe smoking. Although this is a small study, the longitudinal evaluation findings have international relevance and make a useful contribution to the understanding of the impact such interventions can have in addressing one of the few forms of tobacco use that is growing in both developed and developing countries.

**Supplementary Information:**

The online version contains supplementary material available at 10.1186/s12889-022-12792-y.

## Background

The dramatic rise in prevalence and geographic spread of waterpipe use (also known as shisha, arghile, nargile, hubbly bubbly) has been described as a “global phenomenon”, and has become more prevalent than cigarette smoking among young people in some Middle Eastern countries [1]. Suggested reasons for this dramatic increase in popularity, predominantly among young people, include the introduction of flavoured tobacco, widespread dissemination via social media, and frequent uncertainty around regulation and enforcement [1, 2].

Waterpipe use is particularly popular with Arabic speaking young people in North America, Europe and other western countries [1, 3]. In the United States, for example, a 2018 study estimated that 480,000 high school students and 150,000 middle school students used waterpipe in the past 30 days [4]. Among US adults, 16.4% were reported to have ever used a waterpipe to smoke tobacco, and of daily or weekly users, 66% were young adults (18-24 years) [5]. In Australia, waterpipe use accounts for a relatively small proportion of tobacco use, with 2.5% of people 14 years and older using waterpipes to smoke tobacco; [6] however, rates are much higher among Australian people of Arabic speaking background. A 2004 survey of Arabic speakers in Sydney reported that 11.4% of respondents used waterpipes and that 1% were daily users; [7] while a 2010 survey of Arabic speakers in Melbourne found that 38% of respondents had smoked a waterpipe, with 4% reporting daily use [8]. As is common elsewhere, [1] waterpipe use among Arabic speakers in Australia has powerful social and cultural dimensions, [2] and there is considerable skepticism regarding potential health risks, and a belief that it is less harmful than cigarette smoking [2].

The perception that waterpipe smoking is not harmful is a dangerous misconception that ignores related health risks, of both direct use and secondary exposure to waterpipe smoke, and discounts addiction. Studies have found that waterpipe smoking is associated with emphysema, chronic obstructive pulmonary disease, coronary artery disease and oesophageal, gastric and lung cancer [9]. Further, the social nature and communal use of waterpipes have been linked to the transmission of a range of infections, such as respiratory viruses, [10] and are “ideal for transmission and may exacerbate the risk for severe COVID-19 through shared use” [11].

The growing research into waterpipe use has primarily focused on prevalence, toxins and health effects, but there has been relatively little analysis on the effectiveness of health promotion interventions targeting waterpipe smoking. A scoping review of health promotion interventions targeting waterpipe smoking found only 10 published intervention studies – 5 policy interventions, 3 web-based educational interventions, 1 behavioural intervention, and only 1 community-level awareness campaign; [12] while a systematic review found only 3 controlled trials – 2 individual behavioural interventions, and 1 community-level intervention [13].

Given the lack of evidence-based interventions targeting waterpipe smoking, the ‘*Shisha No Thanks’* project was a novel intervention that drew upon practices that have been used in other areas of tobacco control. The ‘*Shisha No Thanks’* project was a co-design, social marketing health promotion campaign targeting waterpipe smoking among young people of Arabic speaking background in Sydney, Australia. Social marketing is a widely used approach to reduce tobacco use, [14] and key strengths of such interventions include the mix of strategies, targeting of specific audiences, and the ‘client-oriented’ approach [15, 16]. The use of a co-design approach taken for the ‘*Shisha No Thanks’* project aimed to ensure the intervention was culturally appropriate and acceptable.

This study evaluates the effectiveness of the ‘*Shisha No Thanks’* project and contributes to the limited existing research on health promotion interventions aimed at waterpipe users. As the target audience of the project is young adults, who are more difficult to engage in research studies, [17] the evaluation also used a novel method of data collection, which was establishing an ‘SMS community panel’ who responded to evaluation survey questions through weekly SMS correspondence.

## Methods

### The ***Shisha No Thanks*** project

The aims of the *Shisha No Thanks* project were to highlight and raise awareness about the health risks of waterpipe smoking among young people (18-35 years old) from an Arabic speaking background and to encourage discussion around quitting or reducing waterpipe smoking. The project ran from October 2019 to June 2020, predominately in the South East, South West, and Western areas of Sydney, Australia, where there is a higher proportion of people who identify as being of Arabic speaking background. The project was run by a government local health district (South Eastern Sydney Local Health District), in partnership with a community organisation (Lebanese Muslim Association) and was funded by the Cancer Institute NSW (a state government cancer control agency).

*Shisha No Thanks* was a co-design project that involved the project team working closely with the community partner organisation, members of the community, community champions and health professionals to identify the key messages and strategies for the awareness raising campaign. The project team was mindful throughout the entire process to ensure that the campaign was run respectfully towards the community and was culturally appropriate.

**Fig. 1:**
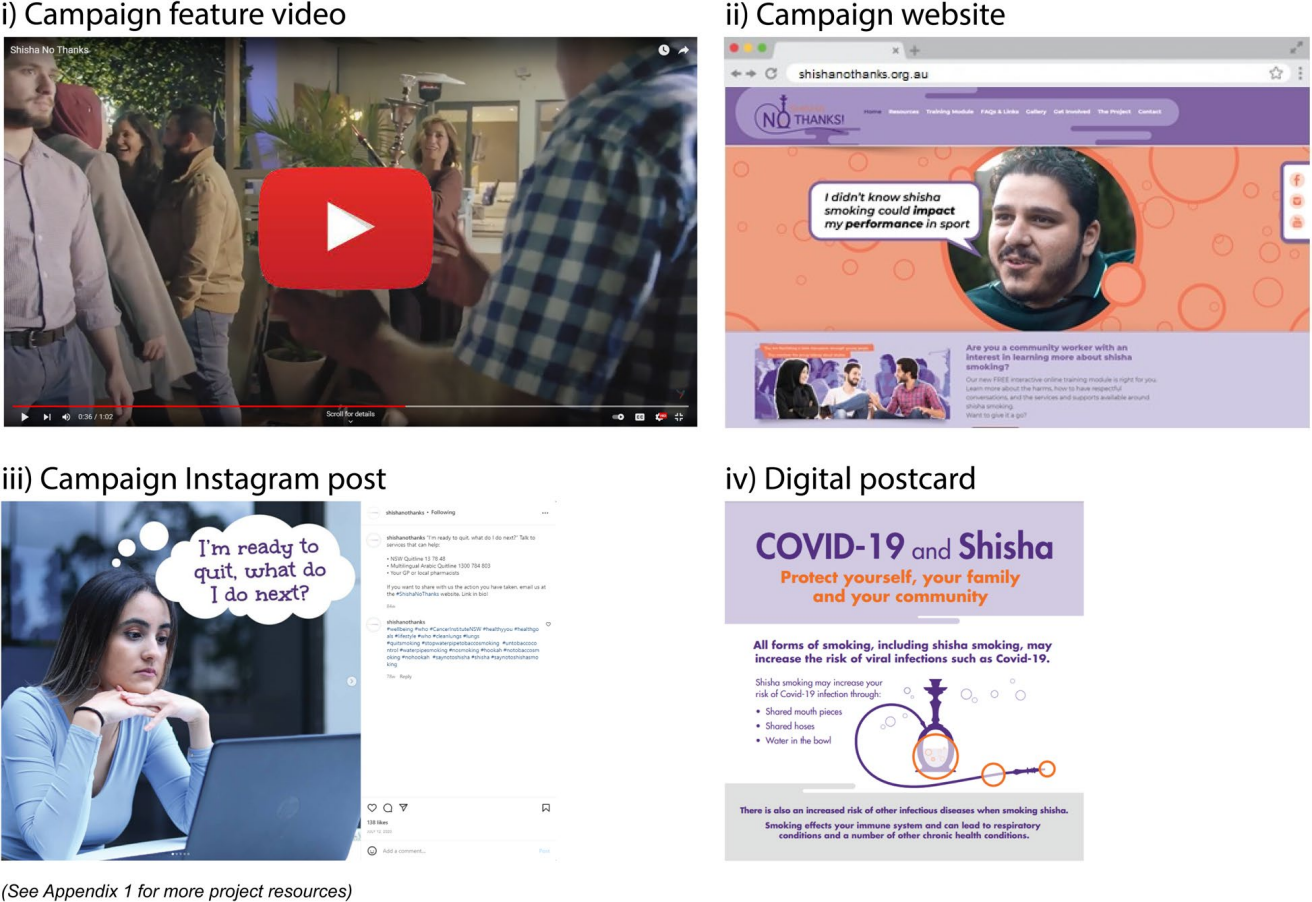
Selected *‘Shisha No Thanks’* project resources

Campaign resources were developed from the community co-design workshops and evidence-based research, and included a feature campaign video, [18] a large collection of social media content (such as short videos clips, memes and graphics), and a suite of factsheets for young people, pregnant women and families, community workers and health professionals, which were available in English and Arabic [19] (See Fig. 1 and Appendix 1 for examples).

These campaign resources were disseminated to the community through the campaign’s website [19] and social media accounts (Facebook, [20] Instagram [21] and YouTube [22]). The project also engaged the community through local media coverage (English and Arabic speaking media; TV, radio and online), by attending community events (e.g. expos and information days) and conducting community worker information sessions.

### Study design, participants and data collection

The impact evaluation used a cohort design to measure awareness before and after the project among the target audience. A community panel was recruited through the Lebanese Muslim Association’s communication channels (email newsletter, social media accounts), community champions, and flyers at events (see Appendix 2 for examples of recruitment material). Participants were required to be 18-35 years old and either smoke waterpipe or know someone who does. Potential participants were directed to complete an online recruitment survey to confirm eligibility in the study, provide demographic details (including their waterpipe smoking activity) and their mobile phone number (See Appendix 3 for Recruitment Survey).

 Participants were then sent a weekly SMS text message with a survey question about their knowledge and attitudes about waterpipe smoking. As most young people use their mobile phones frequently each day, an SMS survey was an effective way of easily reaching the target audience. Participants were sent a set of 8 questions before the project started, with 1 question being sent per week for 8 weeks from Aug-Oct 2019. Then the same 8 questions were sent towards the end of the project, again with 1 question being sent per week for 8 weeks from Jan-Mar 2020. In the interim period, participants were sent other questions related to waterpipe smoking to maintain communication between participants and the project. (See Appendix 4 for Survey Questions). This approach of sending 1 question per week was chosen to reduce the perceived burden of responding to the survey questions. Main participant recruitment documents and all data collection surveys were produced in English and Arabic, and participants were given the option to choose to receive the SMS text messages in either English or Arabic.

### Survey measures

The SMS survey questions were adapted from the Cancer Institute NSW Tobacco Tracking Survey [23] and the Syrian Center for Tobacco Studies Narghile-Waterpipe Users Survey [24]. The questions were related to participants’ awareness of messages about the harms of waterpipe smoking, attitudes towards the health impacts of waterpipe smoking, intention to reduce waterpipe smoking, community conversations about waterpipe smoking, and awareness of services to support cessation of waterpipe smoking. Questions were designed to be short and succinct to fit with the SMS format, and were either multiple choice response, or short free-text response.

Participants were reimbursed for their involvement in the study with three $50AUD e-vouchers. The survey used the Qualtrics platform which has the capacity to send SMS messages to the study participants’ mobile phone number.

### Analysis

Data extracted from Qualtrics was entered into an Excel spreadsheet file. Data was then analysed using IBM SPSS Statistics v26. For the 8 questions that were asked before and after the project, only paired data (i.e. data where the participant had responded to the same question at both baseline and post-campaign) were used for analysis and reported. Given the matched nature of the data, binary categorical responses were analysed using McNemar’s test, [25, 26] and non-parametric scaled data was analysed using Wilcoxon Signed Rank test [27]. Subgroup analysis was also conducted based on age group, gender and waterpipe use. For the 6 questions that were asked only once (in the interim period), descriptive analysis was conducted.

## Results

In total, 133 people were recruited to the study’s SMS community panel (see Table 1). 86 (64.7%) were female, the mean age of the panel was 25.8 years old, and 87 (65.4%) participants reported speaking English and Arabic at home. 100 (75.2%) participants reported smoking waterpipe, with 22 reporting smoking waterpipe daily, 35 smoking waterpipe at least once per week (but not daily), and 37 reporting smoking waterpipe less than once per week. The number of participants who responded to each question both at baseline and post-campaign ranged from 70 to 92 (see Table 2 and Appendix 5).

**Table 1:** Demographic characteristics of SMS panel participants (*n*=133)

		n	%
**Age**			
18-26 years old		80	60.2
27-35 years old		53	39.8
**Gender**			
Male		47	35.3
Female		86	64.7
**Language spoken at home**			
English		28	21.1
Arabic		12	9.0
English and Arabic		87	65.4
Other		6	4.5
**Smoking waterpipe at recruitment**			
Yes		100	75.2
No		32	24.1
Not sure		1	0.8
**Frequency of waterpipe smoking**^**a**^			
Daily		22	16.8
At least once per week, but less than daily		35	26.7
Less than once per week		37	28.2
Not applicable		37	28.2

^a^Data for 2 participants missing

**Fig. 2:**
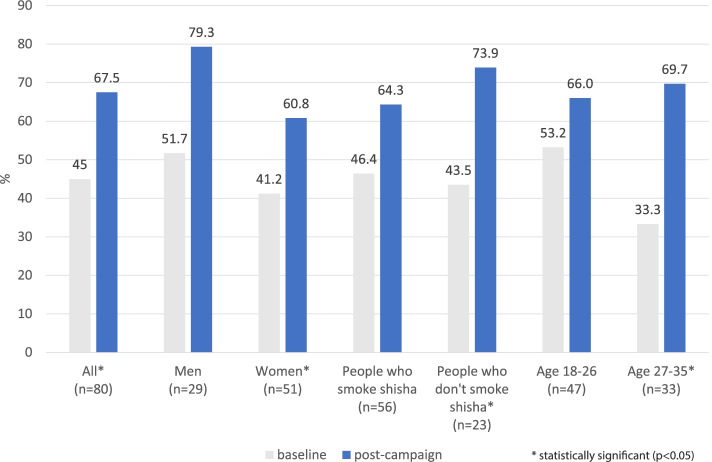
Proportion of people who had seen, heard or read about the harms of shisha smoking

When asked whether they had seen, heard or read anything about the harms of waterpipe smoking, there was an increase in the proportion who reported they had post-campaign (*n*=54, 67.5%) compared with baseline (*n*=36, 45.0%). This is the only statistically significant change identified in this study (*p*=0.003) (see Table 2). In the subgroup analyses, this result was significant among women, people in the older age group (27-35 year olds) and people who did not smoke waterpipe (see Fig. 2 and Appendix 6). When asked to describe what they had seen, heard or read, 34 of the 44 valid responses were consistent with the main messages or resources of the *Shisha No Thanks* project.

**Table 2:** Paired responses at baseline and post-campaign

		Baseline		Post-campaign		*p-value*
		n	**%**	n	**%**	
**Have you seen, heard or read anything about harms of shisha smoking (*n*=80)**						p=0.003*
Yes		36	**45.0**	54	**67.5**	
No or Don’t know		44	**55.0**	26	**32.5**	
**Shisha contains cancer-causing substances (*n*=84)**						*p=0.13*
Strongly agree		36	**42.9**	47	**56.0**	
Somewhat agree		29	**34.5**	20	**23.8**	
Neutral / Don’t know		17	**20.2**	15	**17.9**	
Somewhat disagree		1	**1.2**	1	**1.2**	
Strongly disagree		1	**1.2**	1	**1.2**	
**What are the health effects of smoking shisha compared to cigarettes? (*n*=81)**						*p=0.82*
Same or more harmful		55	**67.9**	53	**65.4**	
Less harmful or Don’t know		26	**32.1**	28	**34.6**	
**Smoking shisha can cause damage to your body (*n*=85)**						*p=0.31*
Strongly agree		46	**54.1**	52	**61.2**	
Somewhat agree		28	**32.9**	23	**27.1**	
Neutral / Don’t know		9	**10.6**	9	**10.6**	
Somewhat disagree		2	**2.4**	1	**1.2**	
Strongly disagree		0	**0.0**	0	**0.0**	
**Have you thought about reducing the amount of shisha you smoke? (*n*=92)**						*p=0.70*
Yes, [Within the next 30 days/ next 6 months/ completely stopping]		43	**46.7**	46	**50.0**	
No / Don’t know		49	**53.3**	46	**50.0**	
**Have you talked to someone about the harms of smoking shisha? (*n*=70)**						*p=0.05*
Yes		44	**62.9**	34	**48.6**	
No / Don’t know		26	**37.1**	36	**51.4**	
**Do you know where to find information or support to help quit smoking shisha? (*n*=80)**						*p=1.00*
Yes		18	**22.5**	18	**22.5**	
No / Don’t know		62	**77.5**	62	**77.5**	

When asked about the health harms of waterpipe smoking, there was a slightly higher proportion of people who strongly agreed that it could cause physical damage post-campaign; however this result was not statistically significant (see Table 2). Similar results were obtained when asked whether waterpipes contain cancer-causing substances (see Table 2). There were also no statistically significant changes for these questions in the subgroup analyses (see Appendix 6).

There were no statistically significant differences in the proportion of participants who considered reducing or quitting waterpipe smoking before or after the campaign, or the proportion of participants who had talked to someone about the harms of waterpipe smoking. Finally, the proportion of participants who were aware of where to obtain information or support to help quit smoking waterpipe was low both at baseline and post campaign (22.5%) (see Table 2).

The questions that were asked between the baseline and post-campaign survey questions provided insight into behaviours related to waterpipe smoking (see Table 3). 46.7% of respondents reported having searched for information about waterpipe on the internet. Of those who had, 37.0% had searched where to buy or smoke waterpipe and 41.3% had searched about the harms of waterpipe smoking. In terms of location, 55.2% of those who smoked waterpipe reported doing so at home, while 32.8% reported they smoked at a restaurant. Panel members were asked an open-ended question about the reasons they smoke waterpipe. The responses generally related to the social aspects, relaxation or de-stress, enjoying the taste or smell of waterpipe, having fun, the cultural or family aspect, or peer pressure.

Finally, panel members were also asked about whether they smoked other tobacco products. There was strong evidence of an association between waterpipe smoking and smoking of other tobacco products, with 37.0% of people who smoked waterpipe also reporting smoking other tobacco products, compared with 7.1% of non-waterpipe smokers smoking other tobacco products (*p*=0.006) (results are not shown).

**Table 3:** Survey responses for questions about waterpipe smoking-related behaviours

		n	%
**Have you ever searched for information about smoking shisha on the internet (*n*=107)**			
Yes		50	**46.7**
No		53	**49.5**
Not sure		4	**3.7**
**If you have ever searched for information about smoking shisha on the internet, what was it about? (*n*=46)**			
How to smoke shisha		2	**4.3**
Where to buy or smoke shisha		17	**37.0**
What are the harms of smoking shisha		19	**41.3**
How to quit smoking shisha		4	**8.7**
Other		4	**8.7**
**If you smoke shisha, where do you mostly smoke it? (*n*=67)**			
At home		37	**55.2**
At restaurant		22	**32.8**
At a park, or other public area		3	**4.5**
Other		5	**7.5**
**Do you currently smoke cigarettes, pipes or other tobacco products (excluding shisha)? (*n*=101)**			
Yes		29	**28.7**
No		69	**68.3**
Don’t know		3	**3.0**
**How often do you now smoke cigarettes, pipes or other tobacco products (excluding shisha)? (*n*=26)**			
Daily		13	**50.0**
At least weekly (not daily)		4	**15.4**
Less often than weekly		6	**23.1**
Not at all, but I have smoked in the last 12 months		3	**11.5**

## Discussion

By using an SMS community panel, this evaluation study showed that the *Shisha No Thanks* project was able to increase awareness of messages about the harms of waterpipe smoking among the target audience of young adults of Arabic speaking background. This adds to the limited number of studies of interventions addressing waterpipe smoking, and indicates that a co-designed social marketing approach, using social media and community events constitutes an effective strategy to raise awareness of this issue.

This evaluation also identified there is a baseline level of awareness of the harms of waterpipe smoking among young adults. The openness of the panel participants towards health messages on this topic could partly be due to the way participants were recruited, and the co-design approach taken for the development of this project. Given the strong cultural associations of waterpipe smoking, it is recommended that future interventions also work closely with the target audience for the intervention to be broadly accepted by communities [2].

While our subgroup analyses found that the increases in awareness of messages about the harms of waterpipe smoking were only statistically significant among non-smokers, women and the older age group, there were still increases detected in all subgroups (see Appendix 6), and the lack of statistical significance may be due in part to the small sample sizes in the subgroups. However, it would be beneficial for future research to assess whether different campaign dissemination channels and campaign messaging are more effective for specific demographics. For example, identifying whether messages should aim to increase knowledge, target people’s health worries, address image perceptions or challenge social norms, would help inform future campaigns targeted at specific audiences.

In the context of other waterpipe smoking interventions, our results are similar to those of a community-based education and awareness intervention in Egypt [28] that had no impact on waterpipe smoking behaviours, but did have an effect on the awareness of the harms of waterpipe smoking. This is consistent with the literature that the success of health campaigns is increased when run in conjunction with other interventions, [29] and therefore suggests that future waterpipe campaigns need to be part of a multipronged approach that uses several health promotion interventions to address waterpipe smoking [12]. For example, our evaluation showed consistent low levels of awareness of support services for people who would like to quit smoking waterpipe, demonstrating the need for greater provision and promotion of support services for people who would like to reduce or quit waterpipe smoking. Policy interventions, similar to those adopted to regulate use and marketing of conventional cigarettes, including smoke-free laws to manage the popular trend of waterpipe smoking bars and lounges, regulations on flavouring additives, and health warning labels on products and related accessories, are other strategies that should be used together with social marketing campaigns. Increased levels of awareness of harms have been found to improve community attitudes towards waterpipe smoking bans, [30] and social marketing campaigns that increase awareness could support the implementation of such policy measures.

Incorporating waterpipe use into broader tobacco control strategies could lead to more sustained progress in reducing this type of tobacco smoking within both the social and cultural groups in which it has been traditionally popular and the growing trend of waterpipe use among the community at large. The culturally appropriate and research-based resources developed for this campaign can be used by other public health organisations, practitioners and cultural groups who can tailor them for use in other geographical areas.

### Strengths and limitations

To our knowledge, this is one of a limited number of studies that have evaluated the impact of a waterpipe smoking intervention, particularly one with a health promotion ethos [13]. The longitudinal study design is a key strength of this study, along with the satisfactory response rate for each question, despite the prolonged duration of the survey and the perception that young adults are difficult to keep engaged in this type of research. An additional strength is that the survey and all recruitment material, were provided in both English and Arabic, which ensured that people were not excluded from the study based on their primary language.

One limitation of this study is the moderate sample size, which limits its ability to detect small changes, particularly for the subgroups we analysed. However, given the resources available, and the size and nature of the project’s target audience, this was a practical compromise in study design. In addition, only including data that had baseline and post-campaign responses could potentially bias results to people who are more engaged with the topic. As the SMS community panel was recruited through the community partner’s communication channels, it is possible that there was an overlap in the people who participated in the co-design workshops with those who were recruited to the panel, which could account for the high proportion of people who responded that they talked to someone about the harms of waterpipe smoking before the campaign. The questionnaire used in this study also did not assess where people encountered the campaign messages (e.g. social media, community events or information sessions). Finally, an additional limitation of this study is that the use of an SMS survey allowed for only short-response format questions.

## Conclusions

This is one of the first published evaluations of a health promotion intervention targeting young people to address the growing global trend of waterpipe smoking. It makes a timely and important contribution that demonstrates that co-design social marketing campaigns can raise awareness of messages about the harms of waterpipe smoking among young people of Arabic speaking background. While the project was not successful in changing attitudes and intentions to quit waterpipe smoking, longer term campaigns, incorporating lessons from other areas of tobacco control could be used to address the growing popularity of waterpipe smoking.

## Supplementary information

**Additional file 1: Appendix 1. **Additional examples of project resources.

**Additional file 2: Appendix 2. **Recruitment material. 

**Additional file 3: Appendix 3. **Recruitment survey questions.

**Additional file 4: Appendix 4. **Survey questions.

**Additional file 5: Appendix 5. **Number of responses for before-after questions. 

**Additional file 6: Appendix 6. **Subgroup Analysis. 

## Data Availability

The dataset used and analysed during the current study are available from the corresponding author on reasonable request.
