# Supplementary figures and images for: Evaluation of ‘Shisha No Thanks’ – a co-design social marketing campaign on the harms of waterpipe smoking
Source: BMC Public Health. 2022 Feb 24;22:386. doi: 10.1186/s12889-022-12792-y (PMC8866041; doi:10.1186/s12889-022-12792-y)

# SUPPLEMENTAL MATERIAL

## Appendix 1 – Additional examples of project resources

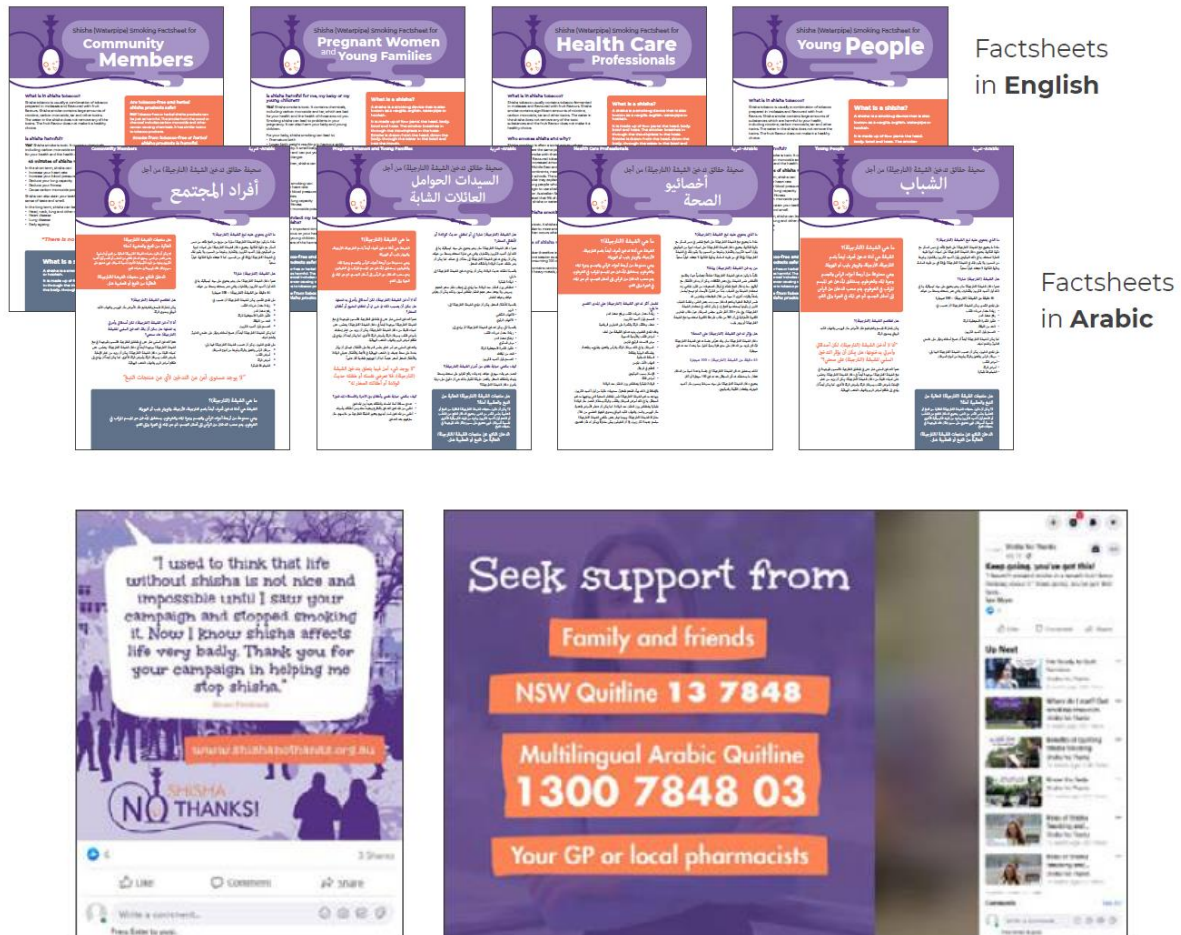

Supplement: Supplementary file 1 — Additional file 1: Appendix 1. Additional examples of project resources. [file 12889_2022_12792_MOESM1_ESM.pdf]
