# Supplementary material for: Evaluation of ‘Shisha No Thanks’ – a co-design social marketing campaign on the harms of waterpipe smoking
Source: BMC Public Health. 2022 Feb 24;22:386. doi: 10.1186/s12889-022-12792-y (PMC8866041; doi:10.1186/s12889-022-12792-y)
Supplement: Supplementary file 2 — Additional file 2: Appendix 2. Recruitment material. [file 12889_2022_12792_MOESM2_ESM.pdf]

SUPPLEMENTAL MATERIAL

Appendix 2 – Recruitment material

Flyer at local events

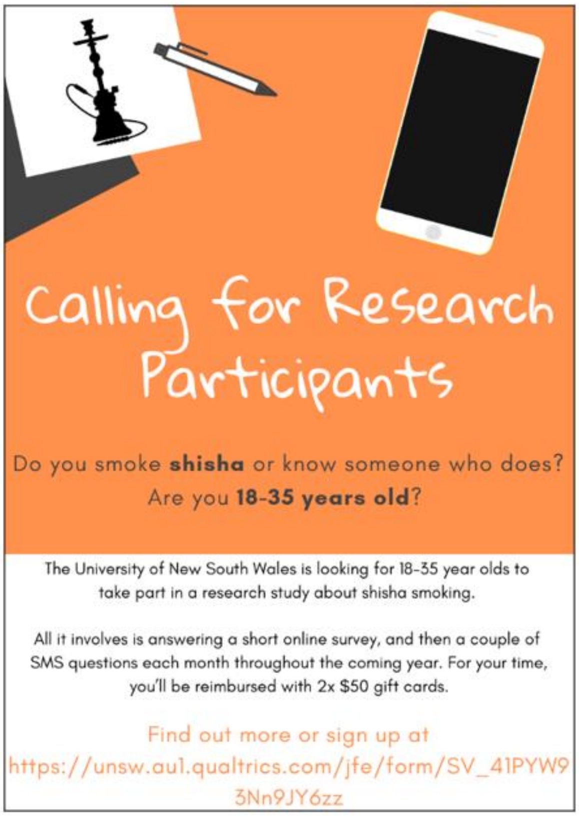

Social media post

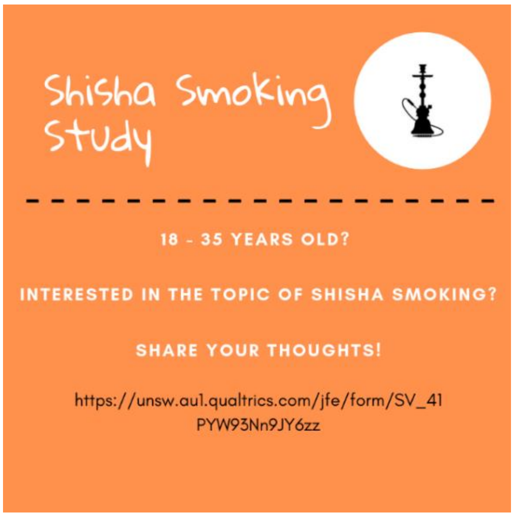

We're looking for 18-35 year olds to take part in a research study on shisha smoking. All it involves is a couple of SMS questions each month. For your time, you will receive 2x $50 e-gift cards from an Australian store. Sign up at https://unsw.au1.qualtrics.com/jfe/form/SV_41PYW93Nn9JY6zz.

#shisha #nargile #hookah #recruiting #health #haveyoursay

Note: Recruitment material stated that participants would receive 2 x $50 e-gift cards as reimbursement for their time. However, this was increased to 3x $50 e-gift cards during the study to increase retention rates for the post-campaign data collection.
