# Supplementary material for: Evaluation of ‘Shisha No Thanks’ – a co-design social marketing campaign on the harms of waterpipe smoking
Source: BMC Public Health. 2022 Feb 24;22:386. doi: 10.1186/s12889-022-12792-y (PMC8866041; doi:10.1186/s12889-022-12792-y)
Supplement: Supplementary file 3 — Additional file 3: Appendix 3. Recruitment survey questions. [file 12889_2022_12792_MOESM3_ESM.pdf]

SUPPLEMENTAL MATERIAL

Appendix 3 - Recruitment survey questions

Question/ Text	Responses
Page 1	
Q1. Would you prefer to complete this survey in English or Arabic?<br>هل تفضل إكمال هذا الاستبيان باللغة الإنجليزية أو العربية؟	○ English<br>○ عربى (Arabic)
Page 2	
Online Participant Information Statement (Appendix 3) and Consent Form (Appendix 4)	
Q2. Do you consent to being contacted with via SMS with 2 survey questions each month over the next 13 months, and for your de-identified information to be used in an evaluation study?	Tick box (compulsory question)
Page 3	
Q3. To ensure you are eligible for this study, how old are you?	Free text response.<br>Validate answer – must be between 18 and 35 (compulsory question)
Q4. What is your gender?	○ Male<br>○ Female<br>○ Prefer not to say
Q5. What is your postcode?	Free text response.<br>Validate answer – must be between 0000 – 9999 (compulsory question)
Q6. What language do you speak at home?	○ English<br>○ Arabic<br>○ English and Arabic<br>○ Other (please specify)
Q7. In the past 12 months, have you smoked shisha? (In these questions, by shisha, we also mean arghile, hookah, hubbly bubbly or waterpipe smoking).	○ Yes<br>○ No<br>○ Not sure
Q8. If yes, how often do you currently smoke shisha?	○ Daily<br>○ At least once per week, but less than daily<br>○ Less than once per week<br>○ Not applicable
Q9. Please provide your mobile phone number for subsequent SMS questions:	Free text response.<br>Validate answer – must be between 0 – 9999999999 (compulsory question)
Page 4	
Thanks for participating in this study and for completing the online survey questions. We will be in touch via SMS soon.<br>If you have any further questions about the study, or would like to opt out at any point, please visit the project website: https://bit.ly/2E9gbFl.	
Q10. Would you like to be kept updated about the project's activities via email?<br>If so, please provide your contact email below:<br>(Please note, we will only contact you for the purposes of this project and will not share your phone number or email address with any other parties.)	Free text response.
