# Supplementary material for: Evaluation of ‘Shisha No Thanks’ – a co-design social marketing campaign on the harms of waterpipe smoking
Source: BMC Public Health. 2022 Feb 24;22:386. doi: 10.1186/s12889-022-12792-y (PMC8866041; doi:10.1186/s12889-022-12792-y)
Supplement: Supplementary file 4 — Additional file 4: Appendix 4. Survey questions. [file 12889_2022_12792_MOESM4_ESM.pdf]

SUPPLEMENTAL MATERIAL

Appendix 4 - Survey questions

Time	Question/ Text
Immediately after completing the online recruitment survey	Hi [name], thanks for being part of our research study. We'll be in touch with survey questions soon. For more info visit https://bit.ly/2E9gbFl.
Baseline (pre-project) data collection	
Week 1<br>Aug 2019	Hi, thanks for being part of the waterpipe (shisha) study. To answer the survey questions, just reply with the number that matches your response. Standard messaging rates apply. Text "STOP" to opt out of this survey. T&Cs: https://bit.ly/2E9gbFl<br>Shisha study question 1: Have you recently seen, read or heard anything online, in the media, or your community about the harms of smoking shisha? (E.g. stories, discussions, information or news). It might have been on TV, radio, in magazines or newspapers, on the internet or somewhere else. Reply with a number (1, 2 or 3).<br>1. Yes<br>2. No<br>3. Don't know
Week 2<br>Aug 2019	If you answered yes to the previous shisha question, can you describe in a few words what you saw, read or heard?
Week 3<br>Aug 2019	Shisha study: Don't forget to reply to all questions to receive your x3 $50 vouchers. You will receive the 1st one next week after replying to question 4.<br>Question 3: What do you think of the following statement? Shisha contains cancer-causing substances? Reply with a number (1-6).<br>1. Strongly agree<br>2. Somewhat agree<br>3. Neutral<br>4. Somewhat disagree<br>5. Strongly disagree<br>6. Don't know
Week 4<br>Aug 2019	Shisha study question 4: How would you rate smoking shisha compared to cigarettes considering its health effects? Reply with a number (1-4).<br>1. Same<br>2. Less harmful<br>3. More harmful<br>4. Don't know
Week 4<br>Aug 2019	[For participants who responded to most of the questions so far]<br>You have received a $50 gift card from UNSW Shisha study. Expires 09 Sep 2020*. Message: Thanks for being part of your research study. Here's a $50 e-gift.<br>* Gift card expires 3 years from issue date.
Week 5<br>Sep 2019	Shisha study question 5: What do you think of the following statement? Smoking shisha can cause damage to your body. Reply with a number (1-6).<br>1. Strongly agree<br>2. Somewhat agree<br>3. Neutral<br>4. Somewhat disagree<br>5. Strongly disagree<br>6. Don't know
Week 6<br>Sep 2019	Shisha study question 6: In the past month, have you thought about reducing the amount of shisha you smoke? Reply with a number (1-5).<br>1. Yes, within the next 30 days<br>2. Yes, within the next 6 months

	3. Yes, completely stopping shisha smoking<br>4. No<br>5. Don't know
Week 7<br>Sep 2019	Shisha study question 7: Have you recently talked to someone (e.g. family or friend) about the harms of smoking shisha? Reply with a number (1-3).<br>1. Yes<br>2. No<br>3. Don't know
Week 8<br>Oct 2019	Shisha study question 8: Do you know of any websites or phone services that someone could use to get information or support to help quit smoking shisha? Reply with a number (1-3).<br>1. Yes<br>2. No<br>3. Don't know
Week 8<br>Oct 2019	[For participants who have responded to most of the questions so far]<br>You have received a $50 gift card from UNSW Shisha study. Expires 09 Sep 2020*. Message: Thanks for being part of your research study. Here's a $50 e-gift.<br>* Gift card expires 3 years from issue date.
Campaign launch (15 Oct 2019)	
During project data collection	
Week 12<br>Oct 2019	Shisha study question 9: Have you ever searched for information about smoking shisha on the internet? Reply with a number (1-3).<br>1. Yes<br>2. No<br>3. Not sure
Week 13<br>Oct 2019	Shisha study question 10: If you have ever searched for information about smoking shisha on the internet, what was it about? Reply with a number (1-5).<br>1. How to smoke shisha<br>2. Where to buy or smoke shisha<br>3. What are the harms of smoking shisha<br>4. How to quit smoking shisha<br>5. Other
Week 14<br>Nov 2019	Shisha study question 11: If you smoke shisha, where do you mostly smoke it? Reply with a number (1-5).<br>1. At home<br>2. At restaurant<br>3. At a park, or other public area<br>4. Other<br>5. I don't smoke shisha
Week 15<br>Nov 2019	Shisha study question 12: What's the main reason(s) you smoke shisha? (in a few words)
Week 16<br>Nov 2019	Shisha study question 13: Do you currently smoke cigarettes, pipes or other tobacco products (excluding shisha)? Reply with a number (1-3).<br>1. Yes<br>2. No<br>3. Don't know
Week 17<br>Nov 2019	Shisha study question 14: How often do you now smoke cigarettes, pipes or other tobacco products (excluding shisha)? Reply with a number (1-6).<br>1. Daily<br>2. At least weekly (not daily)<br>3. Less often than weekly<br>4. Not at all, but I have smoked in the last 12 months<br>5. Not at all and I have not smoked in the last 12 months<br>6. Not applicable, I have never smoked

Follow-up data collection	
13 Jan 2020	Shisha study question 1: Have you recently seen, read or heard anything online, in the media, or your community about the harms of smoking shisha? (E.g. stories, discussions, information or news). It might have been on TV, radio, in magazines or newspapers, on the internet or somewhere else. Reply with a number (1, 2 or 3).<br>1. Yes<br>2. No<br>3. Don't know
20 Jan 2020	If you answered yes to the previous shisha question, can you describe in a few words what you saw, read or heard?
28 Jan 2020	Shisha study question 3: What do you think of the following statement? Shisha contains cancer-causing substances. Reply with a number (1-6).<br>1. Strongly agree<br>2. Somewhat agree<br>3. Neutral<br>4. Somewhat disagree<br>5. Strongly disagree<br>Don't know
3 Feb 2020	Shisha study question 4: How would you rate smoking shisha compared to cigarettes considering its health effects? Reply with a number (1-4).<br>1. Same<br>2. Less harmful<br>3. More harmful<br>4. Don't know
10 Feb 2020	Shisha study question 5: What do you think of the following statement? Smoking shisha can cause damage to your body. Reply with a number (1-6).<br>1. Strongly agree<br>2. Somewhat agree<br>3. Neutral<br>4. Somewhat disagree<br>5. Strongly disagree<br>6. Don't know
17 Feb 2020	Shisha study question 6: In the past month, have you thought about reducing the amount of shisha you smoke? Reply with a number (1-5).<br>1. Yes, within the next 30 days<br>2. Yes, within the next 6 months<br>3. Yes, completely stopping shisha smoking<br>4. No<br>5. Don't know
24 Feb 2020	Shisha study question 7: Have you recently talked to someone (e.g. family or friend) about the harms of smoking shisha? Reply with a number (1-3).<br>1. Yes<br>2. No<br>3. Don't know
2 Mar 2020	Shisha study question 8: Do you know of any websites or phone services that someone could use to get information or support to help quit smoking shisha? Reply with a number (1-3).<br>1. Yes<br>2. No<br>3. Don't know
2 Mar 2020	[For participants who have responded to most of the questions so far]<br>You have received a $50 gift card from UNSW Shisha study. Expires 09 Sep 2020*. Message: Thanks for being part of your research study. Here's a $50 e-gift.<br>* Gift card expires 3 years from issue date.

2 Mar 2020	There are no more questions for this research study, and we will not be contacting you via SMS again. If you would like to be informed of the results of the study, please visit http://bit.do/foTef for contact details. Thanks again for being part of the study.
