# Supplementary material for: Evaluation of ‘Shisha No Thanks’ – a co-design social marketing campaign on the harms of waterpipe smoking
Source: BMC Public Health. 2022 Feb 24;22:386. doi: 10.1186/s12889-022-12792-y (PMC8866041; doi:10.1186/s12889-022-12792-y)
Supplement: Supplementary file 5 — Additional file 5: Appendix 5. Number of responses for before-after questions. [file 12889_2022_12792_MOESM5_ESM.pdf]

## SUPPLEMENTAL MATERIAL

## Appendix 5 - Number of responses for before-after questions

| | Baseline (n) | Post-campaign (n) | **Paired data (Both Baseline and Post-campaign) (n)** |
|---|---|---|---|
| Have you seen, heard or read anything about harms of shisha smoking | 101 | 89 | **80** |
| Shisha contains cancer-causing substances | 105 | 87 | **84** |
| What are the health effects of smoking shisha compared to cigarettes? | 103 | 87 | **81** |
| Smoking shisha can cause damage to your body | 112 | 85 | **85** |
| Have you thought about reducing the amount of shisha you smoke? | 106 | 93 | **92** |
| Have you talked to someone about the harms of smoking shisha? | 76 | 93 | **70** |
| Do you know where to find information or support to help quit smoking shisha? | 105 | 87 | **80** |
