# Supplementary material for: Evaluation of ‘Shisha No Thanks’ – a co-design social marketing campaign on the harms of waterpipe smoking
Source: BMC Public Health. 2022 Feb 24;22:386. doi: 10.1186/s12889-022-12792-y (PMC8866041; doi:10.1186/s12889-022-12792-y)
Supplement: Supplementary file 6 — Additional file 6: Appendix 6. Subgroup Analysis. [file 12889_2022_12792_MOESM6_ESM.pdf]

Appendix 6 - Subgroup Analysis

Gender

Men

	Baseline		Post-campaign		*p-value*
	n	%	n	%	
**Have you seen, heard or read anything about harms of shisha smoking (n=29)**					*p=0.06*
Yes	15	**51.7**	23	**79.3**	
No or Don’t know	14	**48.3**	6	**20.7**	
**Shisha contains cancer-causing substances (n=32)**					*p=0.64*
Strongly agree	17	**53.1**	19	**59.4**	
Somewhat agree	10	**31.3**	9	**28.1**	
Neutral / Don’t know	4	**12.5**	3	**9.4**	
Somewhat disagree	1	**3.1**	1	**3.1**	
Strongly disagree	0	**0.0**	0	**0.0**	
**What are the health effects of smoking shisha compared to cigarettes? (n=29)**					*p=0.73*
Same or more harmful	14	**48.3**	16	**55.2**	
Less harmful or Don’t know	15	**51.7**	13	**44.8**	
**Smoking shisha can cause damage to your body (n=31)**					*p=0.31*
Strongly agree	14	**45.2**	19	**61.3**	
Somewhat agree	14	**45.2**	8	**25.8**	
Neutral / Don’t know	2	**6.5**	4	**12.9**	
Somewhat disagree	1	**3.2**	0	**0.0**	
Strongly disagree	0	**0.0**	0	**0.0**	
**Have you thought about reducing the amount of shisha you smoke? (n=35)**					*p=0.42*
Yes, [Within the next 30 days/ next 6 months/ completely stopping]	19	**54.3**	23	**65.7**	
No / Don’t know	16	**45.7**	12	**34.3**	
**Have you talked to someone about the harms of smoking shisha? (n=25)**					*p=0.22*
Yes	16	**64.0**	12	**48.0**	
No / Don’t know	9	**36.0**	13	**52.0**	
**Do you know where to find information or support to help quit smoking shisha? (n=29)**					*p=1.00*
Yes	7	**24.1**	7	**24.1**	
No / Don’t know	22	**75.9**	22	**75.9**	

Women

	Baseline		Post-campaign		*p-value*
	n	**%**	n	**%**	
**Have you seen, heard or read anything about harms of shisha smoking (n=51)**					*p=0.03**
Yes	21	**41.2**	31	**60.8**	
No or Don't know	30	**58.8**	20	**39.2**	
**Shisha contains cancer-causing substances (n=52)**					*p=0.13*
Strongly agree	19	**36.5**	28	**53.8**	
Somewhat agree	19	**36.5**	11	**21.2**	
Neutral / Don't know	13	**25.0**	12	**23.1**	
Somewhat disagree	0	**0.0**	0	**0.0**	
Strongly disagree	1	**1.9**	1	**1.9**	
**What are the health effects of smoking shisha compared to cigarettes? (n=52)**					*p=0.34*
Same or more harmful	41	**78.8**	37	**71.2**	
Less harmful or Don't know	11	**21.2**	15	**28.8**	
**Smoking shisha can cause damage to your body (n=54)**					*p=0.60*
Strongly agree	32	**59.3**	33	**61.1**	
Somewhat agree	14	**25.9**	15	**27.8**	
Neutral / Don't know	7	**13.0**	5	**9.3**	
Somewhat disagree	1	**1.9**	1	**1.9**	
Strongly disagree	0	**0.0**	0	**0.0**	
**Have you thought about reducing the amount of shisha you smoke? (n=57)**					*p=1.00*
Yes, [Within the next 30 days/ next 6 months/ completely stopping]	24	**42.1**	23	**40.4**	
No / Don't know	33	**57.9**	34	**59.6**	
**Have you talked to someone about the harms of smoking shisha? (n=45)**					*p=0.21*
Yes	28	**62.2**	22	**48.9**	
No / Don't know	17	**37.8**	23	**51.1**	
**Do you know where to find information or support to help quit smoking shisha? (n=51)**					*p=1.00*
Yes	11	**21.6**	11	**21.6**	
No / Don't know	40	**78.4**	40	**78.4**	

Age

Younger age group (18-26 years old)

	Baseline n	%	Post-campaign n	%	*p-value*
**Have you seen, heard or read anything about harms of shisha smoking (n=47)**					*p=0.21*
Yes	25	**53.2**	31	**66.0**	
No or Don't know	22	**46.8**	16	**34.0**	
**Shisha contains cancer-causing substances (n=47)**					*p=0.18*
Strongly agree	19	**40.4**	27	**57.4**	
Somewhat agree	16	**34.0**	9	**19.1**	
Neutral / Don't know	11	**23.4**	9	**19.1**	
Somewhat disagree	0	**0.0**	1	**2.1**	
Strongly disagree	1	**2.1**	1	**2.1**	
**What are the health effects of smoking shisha compared to cigarettes? (n=46)**					*p=0.34*
Same or more harmful	30	**65.2**	26	**56.5**	
Less harmful or Don't know	16	**34.8**	20	**43.5**	
**Smoking shisha can cause damage to your body (n=50)**					*p=0.36*
Strongly agree	28	**56.0**	32	**64.0**	
Somewhat agree	14	**28.0**	11	**22.0**	
Neutral / Don't know	7	**14.0**	6	**12.0**	
Somewhat disagree	1	**2.0**	1	**2.0**	
Strongly disagree	0	**0.0**	0	**0.0**	
**Have you thought about reducing the amount of shisha you smoke? (n=54)**					*p=0.63*
Yes, [Within the next 30 days/ next 6 months/ completely stopping]	25	**46.3**	28	**51.9**	
No / Don't know	29	**53.7**	26	**48.1**	
**Have you talked to someone about the harms of smoking shisha? (n=42)**					*p=0.06*
Yes	26	**61.9**	18	**42.9**	
No / Don't know	16	**38.1**	24	**57.1**	
**Do you know where to find information or support to help quit smoking shisha? (n=52)**					*p=1.00*
Yes	14	**26.9**	13	**25.0**	
No / Don't know	38	**73.1**	39	**75.0**	

Older age group (27-35 years old)

	Baseline		Post-campaign		*p-value*
	n	%	n	%	
**Have you seen, heard or read anything about harms of shisha smoking (n=33)**					*p=0.004**
Yes	11	**33.3**	23	**69.7**	
No or Don't know	22	**66.7**	10	**30.3**	
**Shisha contains cancer-causing substances (n=37)**					*p=0.45*
Strongly agree	17	**45.9**	20	**54.1**	
Somewhat agree	13	**35.1**	11	**29.7**	
Neutral / Don't know	6	**16.2**	6	**16.2**	
Somewhat disagree	1	**2.7**	0	**0.0**	
Strongly disagree	0	**0.0**	0	**0.0**	
**What are the health effects of smoking shisha compared to cigarettes? (n=35)**					*p=0.73*
Same or more harmful	25	**71.4**	27	**77.1**	
Less harmful or Don't know	10	**28.6**	8	**22.9**	
**Smoking shisha can cause damage to your body (n=35)**					*p=0.61*
Strongly agree	18	**51.4**	20	**57.1**	
Somewhat agree	14	**40.0**	12	**34.3**	
Neutral / Don't know	2	**5.7**	3	**8.6**	
Somewhat disagree	1	**2.9**	0	**0.0**	
Strongly disagree	0	**0.0**	0	**0.0**	
**Have you thought about reducing the amount of shisha you smoke? (n=38)**					*p=1.00*
Yes, [Within the next 30 days/ next 6 months/ completely stopping]	18	**47.4**	18	**47.4**	
No / Don't know	20	**52.6**	20	**52.6**	
**Have you talked to someone about the harms of smoking shisha? (n=28)**					*p=0.73*
Yes	18	**64.3**	16	**57.1**	
No / Don't know	10	**35.7**	12	**42.9**	
**Do you know where to find information or support to help quit smoking shisha? (n=28)**					*p=1.00*
Yes	4	**14.3**	5	**17.9**	
No / Don't know	24	**85.7**	23	**82.1**	

Shisha smoking status

People who smoke shisha

	Baseline		Post-campaign		*p-value*
	n	**%**	n	**%**	
**Have you seen, heard or read anything about harms of shisha smoking (n=56)**					*p=0.05*
Yes	26	**46.4**	36	**64.3**	
No or Don't know	30	**53.6**	20	**35.7**	
**Shisha contains cancer-causing substances (n=57)**					*p=0.22*
Strongly agree	17	**29.8**	27	**47.4**	
Somewhat agree	23	**40.4**	13	**22.8**	
Neutral / Don't know	16	**28.1**	15	**26.3**	
Somewhat disagree	0	**0.0**	1	**1.8**	
Strongly disagree	1	**1.8**	1	**1.8**	
**What are the health effects of smoking shisha compared to cigarettes? (n=55)**					*p=1.00*
Same or more harmful	34	**61.8**	34	**61.8**	
Less harmful or Don't know	21	**38.2**	21	**38.2**	
**Smoking shisha can cause damage to your body (n=59)**					*p=0.19*
Strongly agree	25	**42.4**	31	**52.5**	
Somewhat agree	23	**39.0**	19	**32.2**	
Neutral / Don't know	9	**15.3**	8	**13.6**	
Somewhat disagree	2	**3.4**	1	**1.7**	
Strongly disagree	0	**0.0**	0	**0.0**	
**Have you thought about reducing the amount of shisha you smoke? (n=65)**					*p=0.52*
Yes, [Within the next 30 days/ next 6 months/ completely stopping]	36	**55.4**	40	**61.5**	
No / Don't know	29	**44.6**	25	**38.5**	
**Have you talked to someone about the harms of smoking shisha? (n=48)**					*p=0.12*
Yes	26	**54.2**	19	**39.6**	
No / Don't know	22	**45.8**	29	**60.4**	
**Do you know where to find information or support to help quit smoking shisha? (n=58)**					*p=1.00*
Yes	12	**20.7**	12	**20.7**	
No / Don't know	46	**79.3**	46	**79.3**	

People who don't smoke shisha

	Baseline		Post-campaign		*p-value*
	n	**%**	n	**%**	
**Have you seen, heard or read anything about harms of shisha smoking (n=23)**					*p=0.04*
Yes	10	**43.5**	17	**73.9**	
No or Don't know	13	**56.5**	6	**26.1**	
**Shisha contains cancer-causing substances (n=26)**					*p=0.41*
Strongly agree	18	**69.2**	19	**73.1**	
Somewhat agree	6	**23.1**	7	**26.9**	
Neutral / Don't know	1	**3.8**	0	**0.0**	
Somewhat disagree	1	**3.8**	0	**0.0**	
Strongly disagree	0	**0.0**	0	**0.0**	
**What are the health effects of smoking shisha compared to cigarettes? (n=26)**					*p=0.73*
Same or more harmful	21	**80.8**	19	**73.1**	
Less harmful or Don't know	5	**19.2**	7	**26.9**	
**Smoking shisha can cause damage to your body (n=26)**					*p=0.74*
Strongly agree	21	**80.8**	21	**80.8**	
Somewhat agree	5	**19.2**	4	**15.4**	
Neutral / Don't know	0	**0.0**	1	**3.8**	
Somewhat disagree	0	**0.0**	0	**0.0**	
Strongly disagree	0	**0.0**	0	**0.0**	
**Have you thought about reducing the amount of shisha you smoke? (n=26)**					*p=1.00*
Yes, [Within the next 30 days/ next 6 months/ completely stopping]	6	**23.1**	5	**19.2**	
No / Don't know	20	**76.9**	21	**80.8**	
**Have you talked to someone about the harms of smoking shisha? (n=21)**					*p=0.45*
Yes	17	**81.0**	14	**66.7**	
No / Don't know	4	**19.0**	7	**33.3**	
**Do you know where to find information or support to help quit smoking shisha? (n=21)**					*p=1.00*
Yes	6	**28.6**	5	**23.8**	
No / Don't know	15	**71.4**	16	**76.2**	
